# Platelet-rich plasma and platelet-derived lipid factors induce different and similar gene expression responses for selected genes related to wound healing in rat dermal wound environment

**DOI:** 10.22099/mbrc.2020.37181.1500

**Published:** 2020-12

**Authors:** Fahri Akbas, Busra Ozdemir, Nurten Bahtiyar, Hulya Arkan, Ilhan Onaran

**Affiliations:** 1 Department of Medical Biology, Faculty of Medicine, Bezmialem Vakif University, Istanbul, Turkey; 2 Department of Biophysics, Cerrahpasa Faculty of Medicine, Istanbul University- Cerrahpasa, Istanbul, Turkey; 3 Department of Medical Biology, Cerrahpasa Faculty of Medicine, Istanbul University-Cerrahpasa, Istanbul, Turkey

**Keywords:** Platelet-rich plasma, Lipid fraction, Wound healing, Gene expression

## Abstract

Although platelet-rich plasma (PRP) is the plasma fraction that contains higher levels of platelet-sequestered proteins such as growth factors and chemokines, it is also abundant in bioactive lipids whose role in wound healing has not been well characterized. This study provides a preliminary evaluation for the effect of the lipid component of PRP on selected genes related to wound healing. Sprague-Dawley rats were classified into four groups after induction of full thickness excisional wounds: the lipid fraction (LF) (lipid extract from PRP) group, PRP group, dimethyl sulfoxide group, and sham group. Subsequently, relevant groups were topically treated with test preparations. Healing wounds were collected on 3rd, 7th and 14th days, and expression levels of 12 genes were determined using qPCR. LF treatment-induced gene expression signature distinct from that induced by PRP treatment, although there are some overlaps in LF- and PRP-responsive genes. Differentially expressed all eight genes (*Cxcl5, Cxc11, Egfr, Tgfb1, IL10, Tgfa, Mmp1, *and* Mmp7*) to LF response were significantly down-regulated at either 3rd, 7th, or 14th days. Also, the comparison between LF- and PRP-treatment groups showed that the LF significantly decreased expression of *Cxcl11, Mmp7, *and* Tgfa* mRNA on day 7 of healing. This study revealed that PRP and its LF induced different and similar gene expression responses of the skin during the repair of full thickness excisional wounds. Identifying mRNA response to LF treatment at whole transcriptome level can be beneficial for comprehensive understanding of the role of platelet-derived lipid factors in wound healing processes.

## INTRODUCTION

Reports on the experiments with various designs support the use of platelet-rich plasma (PRP) to enhance wound healing [1-3]. Its beneﬁcial effects have been solely attributed to platelet-derived growth and bactericidal factors [4]. Platelets as a main component of the PRP, contain more than 1100 different proteins, which can participate in tissue repair and wound healing. Furthermore, stimulated platelets are characterized by a highly active lipid metabolism as well as enzymatic systems producing various bioactive lipids [5, 6]. There is mounting evidence demonstrating that some bioactive lipids in platelets play important roles in skin health as components of structural lipids, precursors of bioactive mediators, signalling molecules and regulators of gene expression [6-8].

Based on the observations that the chronic wound microenvironment involve increased levels of several proteinases, which could have deleterious effects on the ability of various peptide growth factors to function within this environment, and the lipids are very resistant to hydrolytic enzymes [9, 10], it has been proposed that the beneficial effect of PRP on wound healing may be derived from its lipid component [11]. Hoeferlin et al. [11] tested this in an *in vitro* model and they demonstrated a direct role for the peptide–free lipid fraction (LF) of PRP in biological mechanisms related to wound healing. Our previous *in vivo* study also showed that the lipid component of PRP enhanced the healing capacity of skin wounds by positive effects, although not as much as PRP [12]. 

Healing process in the damaged tissue is a very complex process in which networks of cellular and biochemical interactions take place. Today, we know that nearly 100 genes are highly regulated in dermal wound microenvironment following wound damage [13, 14]. Interest in studies of differentially expressed mRNAs of the healing–impaired wounds has also increased in recent years, because it is hoped that such studies may provide important clues for understanding the molecular mechanisms that control the wound repair [15-18].

Given our previous histological findings showing that lipid fraction has a different wound healing capacity compared to PRP, it is natural to hope that the modulation of gene expression by LF may be different from that by PRP in the wound microenvironment. However, its role in modulating gene expression in the skin wound environment during healing has not been evaluated. Therefore, this study aims to evaluate the effect of LF on the expression of wound healing genes in a rat model with full-thickness skin defect by the analysis of the expression of selected 12 genes from previous studies related to PRP treatment. 

## MATERIALS AND METHODS

**Experimental Protocol and Wound Creation: **This experimental study was carried out with 20 adult female Sprague–Dawley rats (weight 200-240 g). All animals were kept under the same environmental conditions, i.e. at a room temperature of 21-24˚C, with an artificial light cycle (lights: 08:00–24:00 h), and were left for one week for adaptation. This experimental study was carried out with the approval of the Bezmialem Vakıf University Experimental Animal Studies Local Ethics Committee, Istanbul, Turkey (no:110/2017). Before the experimental procedures, the rats were anesthetized with ketamine (50–100 mg/kg) and xylazine (10 mg/kg) by intraperitoneal injection. The dorsal skin of the animals was shaved and disinfected using 70% ethanol*.* Then, full-thickness, equidistant, and 12 mm diameter dorsal skin excisions were created. The animals were divided into 4 groups (5 rats per group) as follows: PRP group - PRP treated group, LF group –LF treated group, DMSO group-DMSO applied group as control of LF-treated group, and sham group- not treated group with the agent. Except for the experimental groups, additionally 8 rats were used to obtain the PRP and LF samples. 

**Collection of Blood Samples: **To collect the blood, rat’s chest was opened by surgical method and blood (5-7 mL) was collected via cardiac puncture by using the 18-20 G needle. The blood samples were placed in tubes containing 3.2% sodium citrate.

**Preparation of PRP: **The upper layer of blood (PRP) following centrifugation (400 × *g* for 10 minutes) was transferred to a tube. Then, the PRP sample was centrifuged at 800 × *g* for 10 minutes and platelet poor plasma (PPP) was removed from the upper layer. Platelet count determinations were performed by using Cell-DYN C1600 (Abbott Pharmacuetical Co., Ltd., Lake Bluff, IL, USA), and PPP was added to each sample to provide 1 × 10^6^ platelets/ µL.

**Extraction of LF from PRP: **Some of the PRP samples (1×10^6^ platelets/μL) were used to obtain LF. After activation with 1 U human thrombin and 10 mM CaCl_2,_ these samples were incubated at 37°C for 30 minutes. After incubation, the mixture was centrifuged (1200 × *g* for 15 minutes), and its supernatant was removed and mixed with absolute ethanol in a 1:5 ratio, followed by agitated stirring until homogenous. Then, the supernatant following centrifugation (12000 × *g* for 20 minutes at 4°C) of the mixture The dried lipids dissolved in 25% DMSO were adjusted by UV-spectrophotometry method at 208 nm to have the total amount of lipid in equal concentrations. Prepared samples were kept at 4°C.

**The treatment and biopsy excision: **The wounds were treated with an equal volume (50 µL) of PRP, LF, or DMSO on 0 (wounds creation day), 3, and 7 days after wounding, and left open and undressed. The biopsy samples were taken from the wounds on days 3, 7 and 14, cleaned with isotonic NaCl solution, and stored at -80°C.

**Tissue Handling, RNA Manipulation, and Real-Time Quantitative PCR** (**RT-qPCR): **Total RNA was extracted using a Direct‐zol ™ RNA MiniPrep (Zymo Research Corporation, Irvine, CA, USA) from biopsy samples. Quantity and purity of the RNA aliquots was assessed with ratios of A_260_/A_280_ and A_260_/A_230_ by NanoDrop 2000c UV–Vis Spectrophotometer (Wilmington, USA). Reverse-transcribed cDNA from RNA was generated by a High Capacity cDNA Reverse Transcription Kit (Applied Biosystems, CA, USA) following the supplied protocol. The expression levels of selected 12 RNAs were determined using the RT-qPCR Detection System (Bio-Rad Laboratories, USA). RT‑qPCR was conducted by using the Master Mixes (Qiagen, Hilden, Germany) according to the manufacturer’s protocols. Amplificationconditions for all reactions included an initial denaturation at 95°C for 10 min, and then 40 cycles of denaturation at 95°C for 15 s, annealing at 58°C for 20 s and extension for 20s at 72°C (see Table 1 for primer sequences). The *GAPDH* and *Actb* housekeeping genes were used as an internal control.

**Table 1:** Primer sequences for all primers

	**Gene name**	**Forward primer**	**Reverse primer**
Collagen type I alpha 1 chain	*Col1a1*	CAACCTCAAGAAGTCCCTGC	AGGTGAATCGACTGTTGCCT
Connective tissue growth factor	*Ctgf*	CAAGCTGCCCGGGAAAT	CGGTCCTTGGGCTCATCA
Chemokine (C-X-C motif) ligand 15	*Cxcl15*	GCATTTCTGCTGCTGTTCACAC	GTTAAGCAAACACAGCGTAGCT
Collagen type III alpha 1 chain	*Col3a1*	AGGGAACAACTGATGGTGCTACTG	GACTGCTGTGCCAAAATAAGAGA
C-X-C motif chemokine 11	*Cxcl11*	CGAAGAAAGATCACCAGAGCCA	CCCCCTTTGAACATAACGAAGC
Epidermal growth factor receptor	*Egfr*	GCGTCTCTTGCCGGAATGT	GGCTCACCCTCCAGAAGGTT
Interleukin-10	*Il10*	CAGAGCTCAGGAAACTGCTG	AGGCCTGGTCTTCTTTCAGA
Matrix metallopeptidase 1	*Mmp1*	GCCATTACTCACAACAATCCTC	ACACAATATCACCTTCCTCCTC
Matrix metallopeptidase 7	*Mmp7*	GCAGAAGTTCTTCGGTTT	TCTGCAGTCCCCCAACTA
Transforming growth factor alpha	*Tgfa*	ATGGTCCCCTCGGCTGGA	GCTGCTTCTTCTGGCTGGCA
Transforming growth factor beta-1	*Tgfb1*	GCCCTGGACACCAACTATTGCT	AGGCTCCAAATGTAGGGGCAGG
Angiopoietin-1	*Angpt1*	CAACAACAACAGCATCCTGCA	TGCAAAGGCTGACAAGGTTATG
Actin, beta	*Actb*	CCCGCGAGTACAACCTTCT	CGTCATCCATGGCGAACT
Glyceraldehyde-3-phosphate dehydrogenase	*GAPDH*	TGATTCTACCCACGGCAAGTT	TGATGGGTTTCCCATTGATGA

**Data and statistical analyses: **Relative expressions of selected genes in LF and PRP treated wounds were calculated using the 2^−ΔΔCt^ (Fold change (FC)) method [19]. Firstly, it was calculated ΔCt (ΔCt = Ct selected gene – Ct Housekeeping Gene) values and then, ΔΔCt (ΔΔCt = ΔCt treatment- ΔCt control) values, and finally, FC values by 2^−ΔΔCt^.

The differences of the ΔCt values for each gene between the rat groups were analysed using SPSS software 18.0, version (SPSS Inc., Chicago, IL, USA) with Mann-Whitney U and Kruskal–Wallis tests. The statistical significance was set at p<0.0042 to compensate for multiple testing error (0.05/12 (the number of analysed genes) = 0.0042) and the FC difference was accepted equal or greater than five fold (FC < (2^-5^ = 0.031), or FC>(2^5^=32).

## RESULTS

In this study, mRNA expression changes of selected 12 genes from previous studies related to PRP treatment, were determined in LF and PRP -treated groups at 3, 7, and 14th days after wounding, and were compared between them, and with their control groups (and DMSO or sham).

**Figure 1:**
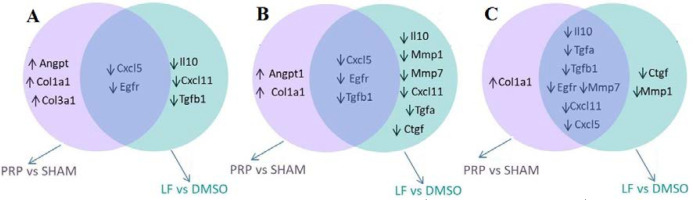
Venn diagram that shows differentially up-regulated ( ) and down-regulated ( ) genes for LF treatment and PRP treatment relative to control wounds (respectively, DMSO and SHAM) in the post-wounding days 3rd (A), 7th (B), and 14th (C). The genes unique to each group is shown inside the circle and the genes changed in two groups is shown in the shaded overlap

**Figure 2:**
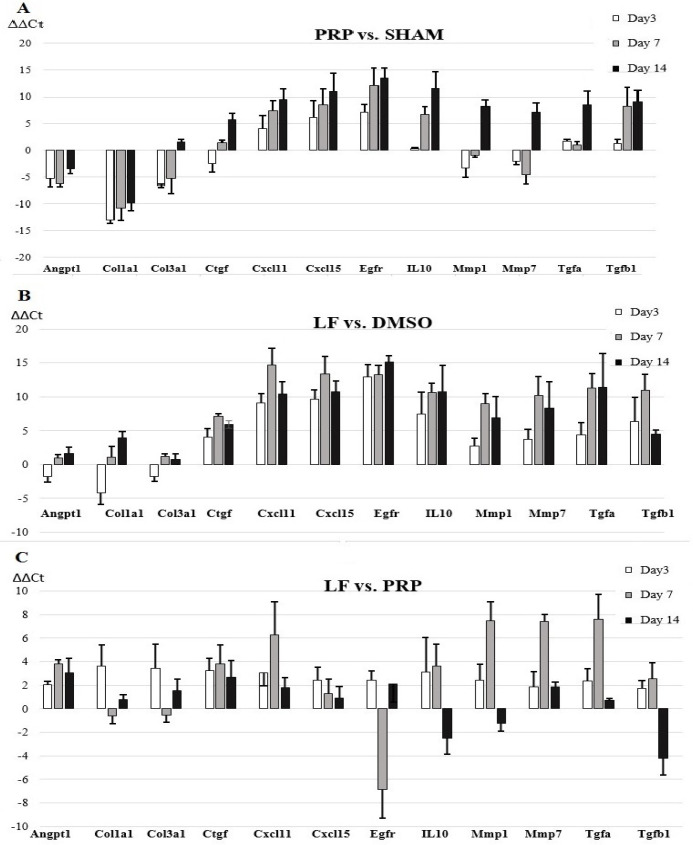
The gene expression level changes of selected 12 RNAs between the groups (PRP vs. Sham (A), LF vs DMSO groups (B), and LF vs PRP (C)) in the post-wounding days 3rd, 7th, and 14th. Data are presented as mean ± standard deviation of the ΔΔCt values

On day 3 after wound damage, the fold regulation analysis indicated that 5 mRNAs in both LF-treated and PRP-treated wounds were differentially expressed at a level of greater than 5-fold in comparison to control wounds. However, the gene expression profile was not the same in both treatment groups. All genes (*Cxcl11, Cxcl15, Egfr, IL10*, and *Tgfb1*) displayed at least a fivefold difference in 3 days were down-regulated in the LF-treated group, while 3 genes (*Angpt1, Col1a1,* and *Col3a1*) and 2 genes (*Cxcl15* and *Egfr*) were upregulated and down-regulated in PRP-treated wounds, respectively. Comparison of the ΔCt values calculated from measuring the treatment and control groups showed that all differentially expressed genes in both treatment groups were significantly different at p<0.0042 (with multiple testing correction) (Table 2). When mRNA gene expression changes were assessed between LF and PRP-treatment groups on 3rd day after wounding, it was seen that there were no significant changes between both groups (Table 3, Fig. 1, Fig. 2).

**Table 2:** The selected 12 mRNA fold changes and the comparisons of LF vs. DMSO and PRP *vs.* SHAM groups at 3, 7, and 14 days

	**LF ** ***vs.*** ** DMSO**			**DMSO ** ***vs.*** ** SHAM**		
	**Day 3**	**Day 7**	**Day 14**	**Day 3**	**Day 7**	**Day 14**
	FC (Mean±SD)	FC (Mean±SD)	FC (Mean±SD)	FC (Mean±SD)	FC (Mean±SD)	FC (Mean±SD)
	**p**	**p**	**p**	**p**	**p**	**p**
***Angpt1***	2.707±0.469	0.985±0.197	0.401±0.061	**36.100** **±6.618**	**71.146** **±12.814**	4.032±0.661
	0.036	0.871	0.008	***0.002***	***0.002***	0.431
***Col1a1***	5.573±0.891	0.719±0.135	0.063±0.011	**7864.288** **±1496.398**	**1723.487±275.940**	**737.185±141.278**
	0.143	0.877	0.007	***0.002***	***0.002***	***0.002***
***Col3a1***	0.898±0.152	0.419±0.079	0.653±0.117	**100.469** **±16.059**	11.269±1.801	1.148±0.218
	0.435	0.038	0.142	***0.002***	0.144	0.877
***Ctgf***	-16.391±3.277	**-142.853** **±22.857**	**-62.016** **±11.007**	5.968±1.004	1.597±0.256	0.050±0.009
	0.007	***0.002***	***0.002***	0.002	0.876	0.005
***Cxcl11***	**-568.181** **±108.527**	**-26775.092** **±5167.389**	**-1374.462** **±230.832**	-19.609±3.009	-27.783±4.439	**-704.225** **±135.213**
	***0.002***	***0.001***	***0.001***	0.002	0.008	***0.002***
***Cxcl15***	**-794.759** **±129.723**	**-10879.423**±**1951.628**	**-1725.927** **±323.362**	**-66.239±12.823**	**-120.106±20.872**	**-1994.457±342.562**
	***0.001***	***0.001***	***0.001***	***0.002***	***0.002***	***0.002***
***Egfr***	-**7727.877±1491.542**	**-10306.633** **±1850.298**	**-36238.319** **±6486.604**	**-138.435±23.976**	**-2047.465±421.838**	**-11048.203±2010.289**
	***0.002***	***0.001***	***0.001***	***0.002***	***0.002***	***0.001***
***IL10***	**-174.655** **±33.009**	**-1626.852** **±317.074**	**-1776.433** **±341.588**	-1.967±0.393	-30.986±5.484	**-2981.850±533.752**
	***0.002***	***0.001***	***0.001***	0.149	0.002	***0.001***
***Mmp1***	-6.774±1.327	**-514.941**±**85.480**	**-119.158** **±20.894**	8.060±1.141	5.518±0.855	0.030±0.006
	0.009	***0.002***	***0.002***	0.030	0.144	0.037
***Mmp7***	-13.131±2.405	**-1195.943** **±233.633**	**-322.913** **±62.43**	2.864±0.514	2.302±0.412	**0.023±0.004**
	0.006	***0.001***	***0.002***	0.147	0.147	***0.003***
***Tgfa***	-21.432±3.486	**-2580.530** **±402.480**	**-2792.531** **±67.173**	0.476±0.092	1.200±0.213	**-363.160±61.502**
	0.005	***0.001***	***0.001***	0.436	0.870	***0.002***
***Tgfb1***	**-80.795** **±14.358**	**-2088.964** **±396.511**	-22.551±3.936	0.330±0.056	**-75.570±1.277**	**-565.641±100.522**
	***0.002***	***0.001***	0.002	0.022	***0.002***	***0.002***

Fold Change values ((FC)=2^-ΔΔCt^) were obtained from triplicate measurement, p values were determined using the ΔCt of both groups. The statistically significance was accepted for fivefold FC (FC<(2^-5^ =0.031), or FC>(2^5^=32)), and p<0.0042. Up- and down-regulated genes were indicated in bold, statistically significant p values were presented in ***bold italic.***

On the 7th day after wounding, there were 9 mRNAs in the LF-treated group (*Ctgf, Cxcl11, Cxcl15, Egfr, IL10, Mmp1, Mmp7, Tgfa, *and* Tgfb1*), and 5 mRNAs in the PRP-treated group (*Angpt1, Col1a1, Cxcl15, Egfr, *and* Tgfb1*) were differentially expressed 5-fold or more in comparison to control wounds. All of the differentially expressed genes were downregulated in the LF-treated group, while 2 of 5 mRNAs (*Angpt1, *and* Col1a1*) were downregulated in the PRP-treated group. In this case, comparison of ΔCt values obtained on the 7th day indicated that all differentially expressed genes passed the Bonferroni-corrected significance threshold of p<0.0042 (Table 2). On the other hand, when LF- and PRP-treatment groups are compared among themselves, the fold change values of 4 mRNAs (C*xcl11, Mmp1, Mmp7, *and* Tgfa*) were downregulated, and only one mRNA (*Egfr*) was upregulated (FC<0.031, or FC>32). However, down-regulated *Mmp1* and upregulated *Egfr* genes with comparison of the ΔCt values did not achieve the p<0.004 level (Table 3, Fig. 1, Fig. 2).

In the 14th day after wounding, although the same number of genes were differentially expressed in the wounds in both treatment groups when compared to their control wounds, their gene expression profile was not the same. The fold regulation analysis showed that 9 mRNAs in both LF-treated and PRP-treated wounds were differentially expressed at a level of greater than 5-fold in comparison to control wounds. All of these 9 mRNAs (*Ctgf, Cxcl11, Cxcl15, Egfr, IL10, Mmp1, Mmp7, Tgfa, *and* Tgfb1*) were downregulated in the LF-treated group, while one mRNA (*Col1a1*) was upregulated, and 8 mRNAs (*Cxcl11, Cxcl15, Egfr, IL10, Mmp1, Mmp7, Tgfa, *and* Tgfb1*) were downregulated in the PRP-treated group (FC<0.031, or FC>32, and p<0.0042). The downregulated *Mmp1* was not to change significantly in the PRP-treated group (p>0.0042) (Table 2). When compared among themselves, there were no significant changes between LF-and PRP-treatment groups on the 14th day after wounding (Table 3, Fig. 1, Fig. 2).

**Table 3:** The selected 12 mRNA fold changes and the comparisons of LF vs. PRP and DMSO *vs.* SHAM groups at 3, 7, and 14 days

	**LF ** ***vs.*** ** PRP**			**DMSO ** ***vs.*** ** SHAM**		
	**Day 3**	**Day 7**	**Day 14**	**Day 3**	**Day 7**	**Day 14**
	FC (Mean±SD)	FC (Mean±SD)	FC (Mean±SD)	FC (Mean±SD)	FC (Mean±SD)	FC (Mean±SD)
	**p**	**p**	**p**	**p**	**p**	**p**
***Angpt1***	4.088±0.755	-14.163±2.553	-1.601±0.302	3.261±0.606	5.107±0.861	6.283±1.123
	0.007	0.012	0.431	0.034	0.004	0.002
***Col1a1***	-12.554±2.246	-2.155±0.421	-1.599±0.296	**113.808±20.294**	**1114.120±198.845**	**9168.584±172.002**
	0.009	0.133	0.437	***0.001***	***0.001***	***0.002***
***Col3a1***	-10.803±2.106	-1.678±0.327	-2.340±0.428	10.351±1.987	15.995±2.476	0.750±0.142
	0.012	0.421	0.149	0.009	0.008	0.870
***Ctgf***	-9.407±1.778	-13.973±2.634	-3.987±0.755	-2.003±0.387	-3.008±0.489	-83.980±15.605
	0.005	0.009	0.142	0.004	0.004	0.879
***Cxcl11***	-2.797±0.465	**-61.144±11.489**	-2.608±0.464	**-66.598±10.494**	**-801.509±142.336**	-1970.672±385.135
	0.421	***0.002***	0.038	***0.002***	***0.002***	0.826
***Cxcl15***	0.870±0.159	0.177±0.033	0.867±0.155	-22.431±3.678	**-48.852±8.976**	-3202.212±601.976
	0.430	0.003	0.032	0.007	***0.005***	0.874
***Egfr***	-5.392±1.028	118.608±20.886	-4.367±0.847	**-466.053±92.734**	**-720.922±120.043**	-681.098±112.348
	0.039	0.439	0.007	***0.002***	***0.003***	0.870
***IL10***	-8.575±4.668	-3.282±0.624	1.260±0.250	-6.368±1.203	**-61.819±10.797**	-3503.085±52.605
	0.008	0.138	0.846	0.005	***0.002***	0.871
***Mmp1***	-5.274±0.954	-177.672±33.323	-4.873±0.912	-2.855±0.536	-30.271±5.712	-83.376±15.405
	0.006	0.834	0.001	0.002	0.009	0.870
***Mmp7***	-3.633±0.645	**-172.174±29.067**	-10.183±1.955	-1.058±0.288	**-60.069±9.595**	-162.488±28.836
	0.141	***0.002***	0.137	0.009	***0.008***	0.864
***Tgfa***	1.013±0.186	**-193.743±34.884**	-10.227±1.688	**-32.388±6.057**	-32.055±6.056	-1190.758±224.056
	0.888	***0.003***	0.033	***0.003***	0.002	0.843
***Tgfb1***	-2.582±0.497	-1.728±0.318	18.835±3.186	-12.275±1.509	-29.513±4.869	-482.503±85.314
	0.149	0.436	0.003	0.002	0.004	0.834

Fold Change values ((FC)=2^-ΔΔCt^) were obtained from triplicate measurement, p values were determined using the ΔCt of both groups. The statistically significance was accepted for fivefold FC (FC < (2^-5^ = 0.031), or FC>(2^5^=32)), and p < 0.0042. Up- and down-regulated genes were indicated in bold, statistically significant p values were presented in ***bold italic.***

## DISCUSSION

The present study provides the first report on a preliminary comparative evaluation with PRP for the effect of the lipid component on the expression of 12 selected genes. Not surprisingly, using more stringent criteria (5-fold cut off and p<0.0042), our data revealed that the LF- and PRP- treatment induced distinct and overlapping expression patterns for the evaluated genes in wound microenvironment during a period of 14 days. Interestingly, all of the differentially expressed genes by the LF treatment were significantly downregulated at either 3, 7, or/and 14 days. In addition, significantly overlapping expression-related genes were also downregulated by both treatments (Fig. 1).

On day 3 after wound damage, three downregulated genes (*Cxcl11, IL10, *and* Tgfb1*) were  identified  as LF-responsive genes. In addition, one of the remarkable points at 3 days was that *Col1a1, Col3a1,* and* Angpt1* mRNA levels were upregulated in the wounds exposed to PRP, but not observed in those with the LF treatment. These results associated with the early stages of wound healing show that the injury environment exposed to the LF reduced the expression of *IL10*, known to be an anti-inflammatory cytokine, and   *TGF-beta*, a growth factor involved in various stages of wound healing. In relation to normal healing process, the expression of *Cxcl11* (an angiostatic chemokine) is quiescent or low at the third day of the skin healing process, which compromises the inflammatory phase in rats [20]. 

The most striking alterations for differently expressed genes with the LF treatment during excisional wound repair were observed at day 7. On this day corresponding to the inflammation and cell proliferation phases, 9 out of a total of 12 genes were downregulated in the LF treatment group, and 3 of these were also found to be downregulated in PRP group, meaning that *Tgfa, Ctgf, Mmp1, Mmp7, IL10,* and *Cxcl11* genes were differentially expressed in response to the LF treatment. The presence of matrix metalloproteinases among LF responsive genes suggests that LF-induced downregulation may not only be limited to suppression of growth factor- and cytokine-related genes (Figure 1 B). We also found that the LF significantly decreased expression of *Cxcl11, Mmp7, *and* Tgfa* mRNAs, as compared to PRP-treated wounds.

On day 14 post wounding, two genes (*Ctg*f and *Mmp1) *were identified as responsive genes to the LF-treatment. Therefore, the observed gene expression changes from the treatment with two different preparations may show that they modulate common and differential pathways and mechanisms in mediating the healing during the repair of full-thickness excisional wounds. Basically, dermal wound microenvironment may exhibit different gene expression responses to the specific combination of growth factors with bioactive lipids, compared with only bioactive lipids.

Since our previous histological findings with same experimental setup showed that the lipid component has a lower healing capacity, we may raise the question of whether down-regulation of these associated genes by LF have an impact on wound healing. Simply, taking into account the generally accepted functions of the associated genes in various stages of the wound healing process, and our results related to the downregulation of several transcript expressions, it can be expected that topically applied LF may cause a negative effect on wound healing than PRP treatment by inhibiting several critical genes for healing. One possible explanation is simply that cellular components and growth factor content of  PRP may exert a better effect for wound healing by synergistically significant promoting effects on the gene expression. In addition, the presence of high levels of certain lipids in LF may also have a negative effect on wound healing compared with PRP by differentially modulating or inhibiting the expression of the associated genes for healing, since Hoeferlin et al. proposed that it may also impair the normal progression of wound repair by manipulating different dynamic processes of the healing [11]. Therefore, further research is needed to explain the regarded gene expression differences between platelet-derived lipid factors- and PRP-treated wounds.

In conclusion, this study showed that and PRP and the lipid component of PRP induced both distinct and different expression patterns for evaluated genes in skin wound environment, suggesting that they could modulate differential and common pathways and mechanisms in mediating wound healing. 
